# Laparoscopic Fertility-Sparing Management of Borderline Ovarian Tumors: Surgical and Long-Term Oncological Outcomes

**DOI:** 10.3390/jcm13185458

**Published:** 2024-09-14

**Authors:** Marta Tortajada Valle, Núria Agustí, Pere Fusté, Eduard Mensión, Berta Díaz-Feijóo, Ariel Glickman, Tiermes Marina, Aureli Torné

**Affiliations:** 1 Gynecology Oncology Unit, Institute Clinic of Gynecology, Obstetrics, and Neonatology, Hospital Clinic of Barcelona, 08036 Barcelona, Spain; 2 Department of Gynecologic Oncology and Reproductive Medicine, The University of Texas MD Anderson Cancer Center, Houston, TX 77030, USA; 3 Institut d’Investigacions Biomèdiques August Pi i Sunyer (IDIBAPS), 08036 Barcelona, Spain; 4 Faculty of Medicine, University of Barcelona, 08036 Barcelona, Spain

**Keywords:** borderline ovarian tumor, survival, laparoscopy

## Abstract

**Objectives:** To assess the long-term oncological safety of laparoscopic fertility-sparing surgery (FSS) in borderline ovarian tumors and the impact of laparoscopic surgical factors on recurrences. Primary outcomes were the recurrence rate and time to recurrence after laparoscopic FSS. Secondary outcomes were to evaluate the recurrence rate after a second laparoscopic surgery and to assess factors associated with the risk of relapse. **Methods:** This is a retrospective single-center observational study in a tertiary university-affiliated hospital. Thirty-four patients diagnosed with borderline ovarian tumors who underwent laparoscopic FSS were recruited. Patients were categorized into two groups: the adnexectomy group, including patients who underwent unilateral adnexectomy, and the cystectomy group, which included patients who underwent unilateral cystectomy, bilateral cystectomy, and unilateral adnexectomy with contralateral cystectomy. **Results:** Eleven relapses (32.3%) were observed during a median follow-up period of 116.1 [62.5–185.4] months. The recurrence rate was similar for patients who underwent cystectomy (6/19, 31.6%) and adnexectomy (5/15, 33.3%). Cystectomy led to a shorter time to first recurrence (36-month progression-free survival rates of 66% vs. 85%) and higher rates of capsular rupture (71.4% vs. 20%, *p* = 0.04) compared to adnexectomy. No deaths due to progression of disease were reported. **Conclusions:** Laparoscopic FSS for borderline ovarian tumors is a safe, long-term oncological option. Although the recurrence rate was similar in patients undergoing adnexectomy or cystectomy, the time to recurrence was shorter in cases treated with cystectomy. Further research is needed to identify eventual laparoscopic risk factors more strongly correlated with recurrence.

## 1. Introduction

Borderline ovarian tumors (BOTs) are tumors of low malignant potential that account for 10–15% of all epithelial malignant tumors. They are diagnosed at an early stage in about 80% of cases and present a better prognosis than epithelial malignant tumors. They are generally diagnosed at a younger age, with one-third of patients being under 40 years, and often affect women who have not completed childbearing [1,2].

Due to the excellent prognosis of BOTs, there has been a trend toward more conservative management to preserve women’s fertility. Fertility-sparing surgery (FSS) enables the conservation of the uterus and at least part of the ovary [3] and includes unilateral adnexectomy or cystectomy.

Nevertheless, there is controversy regarding surgical approaches for BOTs. Although international guidelines [4] advocate open surgery for precise exploration and to facilitate a reduction in the risk reduction of tumoral rupture, retrospective studies consistently support minimally invasive surgery (MIS) as a safe option offering less morbidity [5,6]. Along this line, it is accepted that if it is possible to perform surgery in early stages without the risk of tumor rupture, laparoscopy with protected extraction is preferred instead of laparotomy [7].

When discussing fertility preservation, the MIS approach is favored due to its potential to minimize adhesion formation and its associated negative impact on fecundity [8]. However, an essential aspect warranting careful consideration is the occurrence of late relapses of BOTs. Few studies on the management of BOTs are available, and these have some limitations, such as a small number of patients with relatively short follow-up periods [5,6,8,9], thereby limiting comprehensive understanding of the long-term outcomes of MIS–FSS in BOT management. Additionally, these studies have not systematically investigated the potential inherit risks of MIS, such as the risk of tumor rupture during surgery, the occurrence of port-site metastases, or its safety in advanced disease stages. As a result, they fail to differentiate the specific role of MIS in disease relapse.

The aim of the present study was to assess the long-term oncological safety of laparoscopy in the FSS treatment of BOTs. Additionally, we aimed to explore the impact of surgical factors that may be associated with recurrence following laparoscopy.

## 2. Methods

This was a single-center observational study that included consecutive patients between January 2001 and December 2020 at the Hospital Clinic of Barcelona. Institutional board approval for the project was obtained previously (HCB/2021/0991).

Patients were identified through the Filemaker database, in which all gynecological surgeons prospectively add all surgical interventions performed within the hospital’s facilities. Eligible patients were those women under 45 years of age diagnosed with BOT who underwent FSS by laparoscopy. Data were prospectively collected from electronic patient charts, information regarding pre-operative diagnostic tests and surgical procedures, intra- and post-operative data, and follow-up information, including time to relapse and type of surgery performed in cases of recurrence. These data were analyzed retrospectively. We excluded patients with open access surgery, incomplete surgical or medical reports, and women referred with relapses from other centers or in whom the diagnosis was reclassified to another entity after histological analysis in our center.

The surgical approach, whether cystectomy or unilateral adnexectomy, was determined at the discretion of the attending surgeon based on several factors, such as suspicion of malignancy or the complexity of the surgery, while also considering the patient’s reproductive preferences.

## 3. Procedures

Surgery was considered fertility-sparing when at least part of one ovary and the uterus were spared. Conservative ovarian treatment consisted of unilateral cystectomy (UC), bilateral cystectomy (BC), unilateral adnexectomy (UA), or unilateral adnexectomy with contralateral cystectomy (UA + CC). Patients were categorized into two groups: the adnexectomy group (UA) and the cystectomy group (UC, BC, or UA + CC).

Perioperative diagnosis with frozen section analysis and subsequent surgical staging, when a borderline tumor was diagnosed, were performed when the surgery was carried out by the oncological surgical team. If there was no suspicion of malignancy in the pre-operative assessment and the non-oncologic gynecological team performed the surgery, the analysis of the surgical specimen was performed postoperatively.

Intraoperative tumor rupture was considered whenever there was an unintentional spill of tumor content into the peritoneal cavity during tumor manipulation, including cases in which an endobag had been utilized. The use of an endobag has been mandatory since 2010. Prior to this date, endobags were mainly used by the gynecologic oncology team, but not extended to the rest of the surgical team, when operating on a non-suspected malignant tumor that was found to have borderline histology in the definitive analysis. Pelvic and/or paraaortic node sampling was also performed as part of the staging procedure until 2003. Other staging procedures routinely used included omentectomy, peritoneal washes to obtain cytology samples, and peritoneal biopsies. Histological studies of all surgical specimens were performed by expert gynecologic pathologists in accordance with the World Health Organization system [10]. Tumor staging was essentially determined according to the International Federation of Gynecology and Obstetrics (FIGO) criteria [11]. 

Patients were followed every 4 and 6 months during the first and second years, respectively, and annually for 10 years or until loss to follow-up thereafter. Physical examination, transvaginal ultrasound, and tumor marker evaluation were performed in every evaluation. A computerized tomography scan was only performed during follow-up at the discretion of the attending surgeon.

## 4. Outcomes

The primary end point of this study was to evaluate recurrence events, defined as the relapse of the same tumor histology after an apparently complete surgical resection, and the time to recurrence, defined as the time from primary surgery to the diagnosis of recurrence. Within the primary objective, we also aimed to find differences in parameters between patients who underwent an adnexectomy or a cystectomy (the latter was considered when there was ovarian tissue remaining at the tumor site).

The secondary objective of this study was to evaluate the recurrence rate of relapsed BOTs treated with laparoscopy and to investigate potential associations between the risk of relapse and various clinicopathological factors. These factors included the FIGO stage, histology, tumor size, type of FSS performed, presence of intraoperative cyst rupture, occurrence of port-site metastases, and the use of an endobag during the surgical procedure.

### Statistical Analysis

Statistical analyses were performed with STATA v.15.1 in February 2024. The normal distribution of samples was confirmed using the Shapiro–Wilk test. Consequently, continuous variables were compared using the T-test and presented as mean ± standard deviation (SD) for trend and dispersion assessment. Correlations of qualitative variables were performed using Chi-square or Fisher’s exact tests. Logistic regression was used to calculate the odds ratio (OR). Follow-up time and time to recurrence were expressed as median and interquartile range (IQR). Survival probabilities for each surgical approach were constructed using Kaplan–Meier survival estimates and compared by log-rank test. Statistical significance was determined when *p* < 0.05. 

## 5. Results

**Figure 1:**
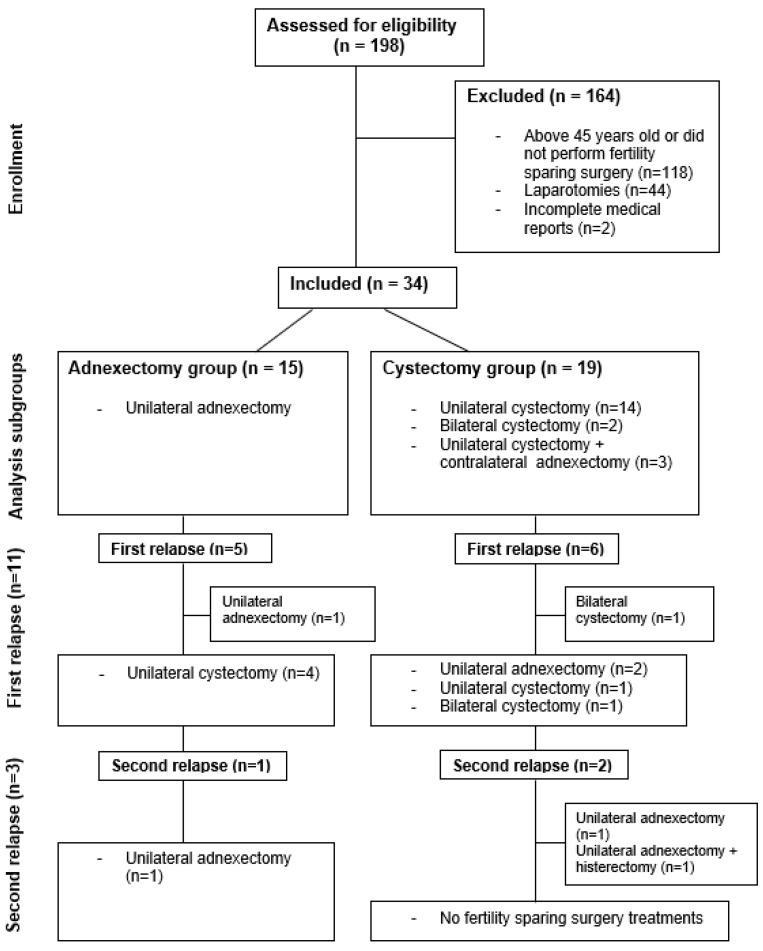
Flowchart of patients presenting relapse following fertility-sparing surgery and conservative treatment.

A total of 198 patients with BOTs were initially assessed (Figure 1). Of these, 118 were women over 45 years of age or who did not undergo FSS, 44 underwent laparotomies, and 2 were excluded because of incomplete medical reports.

Finally, a total of 34 patients who met the inclusion criteria were included. Among them, 15 underwent adnexectomy and 19 underwent cystectomy. Within the cystectomy group, 14 patients underwent UC, 2 patients underwent BC, and 3 patients underwent UC + CA.

### 5.1. Clinical and Surgical Characteristics

The mean age at surgery was 32.7 years (SD 5.9). None of the patients were in menopause. The mean tumor size was 8.8 cm (SD 6.8), with 26 patients (76.5%) having tumors smaller than 10 cm. The serous subtype was the predominant histology in 18 patients (52.9%), followed by mucinous in 10 (29.4%), sero-mucinous in 3 patients (8.8%), endometrioid in 2 patients (5.9%), and clear cells in 1 patient (2.9%). There were no significant differences between the two groups (*p* = 0.7). FIGO stages IA–IB were diagnosed in 12 patients (35.3%), and stages IC–II in 22 patients (64.7%).

Bilateral tumors were diagnosed in five patients (14.7%), and in all these cases at least a cystectomy was performed to preserve fertility. Tumor capsular rupture occurred in 18 patients (52.9%), comprising 3 out of 15 (20%) adnexectomies and 15 out of 19 (78.9%) cystectomies (*p* < 0.001). Extraction of the cyst was performed with an endobag in 18 patients (52.9%).

No intraoperative complications were recorded. Four postoperative complications occurred (11.8%): two postoperative hemorrhages and one trocar hernia that required re-intervention (all classified as Clavien–Dindo IIIB), along with one surgical wound infection managed with conservative treatment (Clavien–Dindo I).

Table 1 summarizes the clinical and surgical characteristics of the patients studied and compares the characteristics of the adnexectomy and cystectomy groups. Univariate analysis of the patients’ characteristics only demonstrated statistically significant differences between the two groups in tumor location and capsular rupture.

### 5.2. Oncologic Outcomes

The total median follow-up duration was 116.1 [62.5–185.4] months. The median follow-up time was 84.4 [47.0–185.4] months in the adnexectomy group and 125 [98.2–187.8] months in the cystectomy group. A total of 11 patients (32.3%) developed tumor recurrence during the follow-up period: five recurrences occurred in the adnexectomy group after a median time from the first surgery of 42.1 [21.1–69.3] months, while six were observed in the cystectomy group (two occurred in patients who underwent BCs and four in patients who underwent UCs) after a median time from the first surgery of 17.9 [10.0–27.6] months.

**Figure 2:**
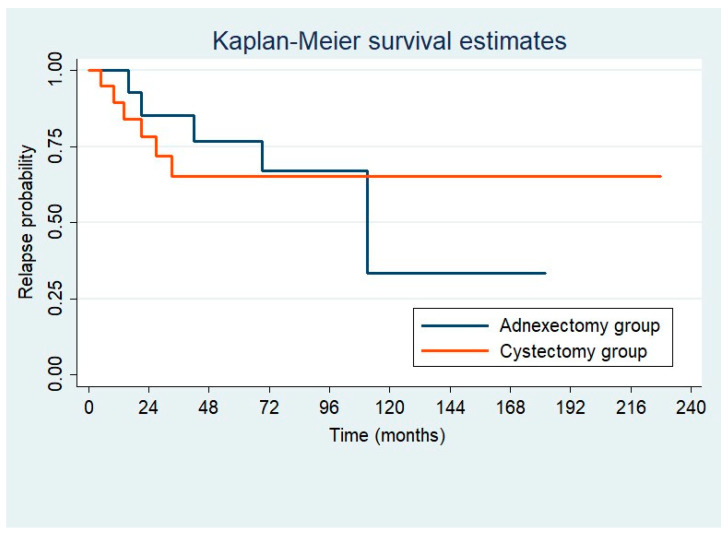
Relapse probability following first surgery on comparing adnexectomy versus cystectomy groups.

Progression-free survival rates after 36 months were 85% (95% CI 0.53–0.96) after adnexectomy and 66% (95% CI 0.39–0.83) after cystectomy. The corresponding 60-month free survival rates were 76% (95% CI 0.32–0.86) for adnexectomy and 66% (95% CI 0.39–0.83) for cystectomy. We did not find differences in the recurrence rate between the groups (cystectomy, 31.6% vs. adnexectomy, 33.3%; *p* = 0.996). Kaplan–Meier estimates of disease-free survival are shown in Figure 2.

No patients with port-site metastases were reported in the study cohort. All recurrences were detected within the remaining ovarian tissue, and all exhibited borderline histology. Of note, all recurrences, except for one, were identified in the same ovary in which the cystectomy had been performed (Supplemental Table S1).

### 5.3. Recurrence Treatment

Nine of the eleven patients (82%) presenting recurrence underwent a second FSS. Specifically, six patients received a UC, two underwent UA, and one underwent a BC. Of these, three patients (33.3%) developed a second relapse (two patients had previously undergone cystectomy during the first relapse surgery, while one patient had undergone UA). For the treatment of the second relapse, one of the patients underwent another laparoscopic FSS. The median follow-up duration between the first and second relapse was 51.0 [12.1–76.3] months. As of the present analysis, all patients were reported to be alive with no evidence of disease (Supplemental Table S2).

Not only were there no relapses in tumors larger than 10 cm, but also the mean size of tumors presenting relapse was significantly smaller than that of those that did not relapse (5.6 cm vs. 10.3 cm, respectively, *p* = 0.008). Regarding histology, FIGO stages and types of surgery performed on the ovary were not significantly different, with ORs of 4 (CI 0.4–41.5), 0.5 (CI 0.1–2), and 0.92 (0.2–3.9), respectively (Table 2).

## 6. Discussion

The present study reports a recurrence rate of 32.3% following laparoscopic FSS over a median follow-up period of 116.1 months. Interestingly, patients in the cystectomy group had a higher incidence of capsular rupture and exhibited a shorter time to first recurrence. However, these factors did not significantly affect the recurrence rate.

Recurrence rates after laparoscopic conservative treatment in the literature vary widely, ranging from 4% to 63% [5,12,13,14,15,16,17] (Table 3), while rates following laparotomy conservative treatment vary between 5% and 37% [18]. The latest meta-analysis comparing laparoscopy and laparotomy [18] did not find a significant difference in the risk of relapse between these two approaches, although it only included retrospective studies with limited follow-up periods.

Our study stands out as having one of the longest follow-up durations reported in the literature, potentially explaining the higher recurrence rate observed. We showed progression-free survival rates for cystectomy and adnexectomy of 66% and 85%, respectively, at 36 months, and 66% and 76%, respectively, at 60 months (Figure 2). Notably, the cystectomy group exhibited a shorter time to first recurrence compared to the adnexectomy group, although the total recurrence rate at the end of the follow-up was similar between both groups. Consistent with the available literature, the majority of recurrences occurred within the first years after surgery. However, the observation of a median time to first recurrence of 42.1 months after adnexectomy in the present study suggests that some previous studies with shorter follow-up periods might have missed the detection of later recurrences. The only randomized trial available was conducted by Palomba et al. [17], who aimed to compare laparoscopic fertility-sparing treatment for BOTs with bilateral tumors and reported a relapse rate of 63%. Although no significant differences in terms of cumulative recurrence rates were detected between the cystectomy and adnexectomy groups, BC resulted in a statistically significant earlier time to relapse. Interestingly, the study by Palomba also had one of the longest follow-ups described, with a median duration of 132 months. Recurrences have been reported in the literature even decades after the initial diagnosis, underscoring the importance of longer follow-up periods [19,20]. Consequently, studies with lower recurrence rates might be associated with shorter follow-up periods [5,12,14,15,16].

When comparing the two types of surgery, patients undergoing cystectomy showed a shorter time to first recurrence in contrast to adnexectomy, aligning with analogous sub-analyses in other studies [17,21]. The presence of residual tumoral cells within the ovary post-cystectomy likely underlies this outcome, although statistical significance was not achieved, probably in relation to the limited sample size. It is worth noting that all recurrences were found in the remaining ovarian tissue with borderline histology, which aligns with previous findings [16,22]. Despite this observed difference, our study, in agreement with published evidence [16,23], did not observe any significant differences in terms of recurrence rate or overall survival rate between patients undergoing adnexectomy or cystectomy. These results suggest that cystectomy remains a valuable and safe approach, even for the management of large or bilateral tumors, without worsening the overall prognosis.

Previous studies suggest that serous tumors present higher rates of recurrence compared to other histological types [18,24]. Although our study observed a lower recurrence rate in tumors with mucinous histology, this difference was not statistically significant. Notably, tumors larger than 10 cm showed no relapses, the majority of which were of mucinous histology. Regarding the occurrence of port-site metastasis, while some studies have linked laparoscopic gas insufflation to such events [25,26,27,28], we did not report any cases of port-site metastases, which is consistent with findings by other research series [12,17]. Several factors may account for this, including the use of endobags, the high proportion of patients diagnosed at an early stage, and the biological characteristics of the tumor. Additionally, we observed intraoperative capsule rupture in half of the procedures performed, a rate in line with what has been reported in other research series [12,13,15]. Notably, most of these ruptures occurred during cystectomies, suggesting that cyst rupture may be more closely associated with the type of surgery.

Up to 82% of recurrences were successfully treated with laparoscopic conservative surgery, and no deaths due to disease progression were observed. Moreover, there was a low rate of complications. These findings highlight the potential of laparoscopic FSS in preserving fertility for patients with BOTs who desire to maintain their childbearing ability [28]. While the decision to complete non-conservative surgery after achieving pregnancy is still under review, some studies [15,29] have demonstrated long periods free of disease solely with conservative approaches. These findings suggest that despite the development of recurrence and the need for subsequent interventions, the overall survival of patients was favorable, with no evidence of active disease at the time of assessment.

One of the main strengths of our study is that it had one of the longest follow-up periods compared to similar studies, a crucial factor considering the prolonged median time to recurrence for BOTs. Moreover, it is one of the few studies that focused exclusively on conservative surgery performed solely via laparoscopy and on risk factors associated with laparoscopy that could compromise long-term oncological outcomes. However, it had certain limitations that need to be acknowledged. Firstly, this study had a retrospective design, which might have introduced some bias. However, this is the only type of study that can be conducted on this topic, as it requires an extended follow-up period that other types of studies cannot accommodate. Nevertheless, as it was a single-center study, the treatment and follow-up were consistent with the protocols of our hospital, resulting in homogeneity across the sample and enhancing the internal validity of the findings. Additionally, the sample size might not have provided sufficient statistical power to obtain significant results. However, this limitation is inherent to our study’s focus on laparoscopic FSS and identifying specific laparoscopic factors.

## 7. Conclusions

Laparoscopic FSS in patients with BOTs is a feasible and safe oncological option. About one third of the patients presented long-term recurrences with no differences between those who underwent cystectomy or adnexectomy. However, cystectomy might be linked to a shorter time to recurrence. Additional investigations are warranted to identify the laparoscopic risk factors most strongly associated with recurrence.

## Figures and Tables

**Table 1:** Comparison of clinical and surgical characteristics between adnexectomy and cystectomy groups.

	ALL	ADNEXECTOMY	CYSTECTOMY	*p*-Value
**Patients, *n (%)***	**34**	**15 (44.1%)**	**19 (55.9%)**	
**Age, *years (mean ± SD)***	**32.7 ± 5.9**	**34.5 ± 6.0**	**31.1 ± 5.4**	**0.08**
**Menopause, *n (%)***				NA
Yes	0 (0%)	0 (0%)	0 (0%)	
No	34 (100%)	15 (100%)	19 (100%)	
**Histology, *n (%)***				**0.7**
Serous	18 (52.9%)	9 (60.0%)	9 (47.4%)	
Mucinous	10 (29.4%)	4 (26.7%)	6 (31.6%)	
Others	6 (17.7%)	2 (13.3%)	4 (21.0%)	
**FIGO stage, *n (%)***				**0.19**
IA–IB	12 (35.3%)	7 (46.7%)	5 (26.3%)	
IC–II	22 (64.7%)	8 (53.3%)	14 (73.7%)	
**Size, *cm (mean ± SD)***	**8.8 ± 6.8 cm**	**10.2 ± 9.4 cm**	**7.7 ± 3.6 cm**	**0.71**
<10 cm	26 (76.5%)	11 (73.3%)	15 (78.9%)	
>10 cm	8 (23.5%)	4 (26.7%)	4 (21.1%)	
**Location, *n (%)***				**0.04**
Unilateral	29 (85.2%)	15 (100%)	14 (73.7%)	
Bilateral	5 (14.7%)	0 (0.0%)	5 (26.3%)	
**Diagnosis, *n (%)***				**0.07**
Perioperative	19 (55.9%)	11 (73.3%)	8 (42.1%)	
Postoperative	15 (44.1%)	4 (26.7%)	11 (57.9%)	
**Capsular rupture, *n (%)***				**<0.001**
Yes	18 (52.9%)	3 (42.9%)	15 (55.6%)	
No	16 (47.1%)	12 (57.1%)	4 (44.4%)	
**Endobag use, *n (%)***				**0.35**
Yes	18 (52.9%)	9 (60%)	9 (47.4%)	
No	16 (47.1%)	6 (40%)	10 (52.6%)	
***Staging, n (%)***				**0.009**
Yes	14 (41.2%)	10 (66.7%)	4 (21.1%)	
No	20 (58.8%)	5 (33.3%)	15 (78.9%)	
**Surgical complications, *n (%)***				**0.40**
Yes	4 (11.8%)	1 (6.7%)	3 (15.8%)	
No	30 (88.2%)	14 (93.3%)	16 (84.2%)	

**Table 2:** Univariate analysis for relapse of clinical and surgical factors.

	Relapse	No Relapse	OR (95% CI)	*p* Value
**Age, *years (mean ± SD)***	32.2 ± 5.8	32.7 ± 6.0	0.9 (0.9–1.1)	0.78
**Histology, *n (%)***				0.26
Serous	8 (72.7%)	10 (43.5%)	4.0 (0.4–41.5)	
Mucinous	2 (18.2%)	8 (34.8%)	1.25 (0.9–17.7)	
Seromucinous	1 (9.1%)	5 (21.7%)		
**FIGO stage, *n (%)***				0.39
IA–IB	5 (45.5%)	7 (30.4%)	0.5 (0.1–2.3)	
IC–II	6 (54.5%)	16 (69.6%)	–	
**Size, *cm (mean ± SD)***	5.6 ± 1.4	10.3 ± 7.8	0.69 (0.4–1.1)	0.008
<10 cm	11 (100%)	15 (65.2%)	NA	
>10 cm	0 (0.0%)	8 (34.8%)	–	
Type of surgery				0.91
Adnexectomy	5 (45.4%)	10 (43.5%)	0.92 (0.2–3.9)	
Cystectomy	6 (54.6%)	13 (56.5%)	–	
**Location, *n (%)***				**0.53**
Unilateral	9 (81.8%)	20 (87.0%)	1.5 (0.2–10.5)	
Bilateral	2 (19.2%)	3 (23.0%)	–	
**Capsular rupture, *n (%)***				**0.59**
Yes	6 (54.6%)	12 (52.2%)	1.1 (0.3–4.6)	
No	5 (45.4%)	11 (47.8%)	–	
**Endobag use, *n (%)***				**0.89**
Yes	6 (54.6%)	12 (52.2%)	1.1 (0.3–4.7)	
No	5 (45.4%)	11 (47.8%)	–	
***Staging, n (%)***				**0.69**
Yes	4 (36.4%)	10 (43.5%)	0.7 (0.2–3.3)	
No	7 (53.6%)	13 (56.5%)	–	

NA: Not applicable.

**Table 3:** Published studies of laparoscopic FSS of BOTs.

	Maneo [13] (n = 30)	Camatte [15] (n = 23)	Fauvet [12] (n = 75)	Palomba [17] (n = 32)	Tinelli [14] (n = 43)	Park [5] (n = 48)	Song [16] (n = 50)	Current Study (n = 34)
**Follow-up, *months (mean/median, IR/SD)***	61 [48–91] *	45 [6–228] *	27.5 ± 33.6 **	132 [117–152] *	44.5 [4–125] *	60 [3–216] *	56 [25.7–80.8] *	116.1 [62.5–185.4] *
**Histology, %** Serous Mucinous Others	73% 23% 4%	65.2% 21.7% 3%	54.3% 33.7% 12%	90.6% 9.4% 0%	16.2% 83.7% 0%	23.4% 75.% 1.1%	23.9% 68.4% 7.7%	18 (52.9%) 10 (29.4%) 6 (17.7%)
**FIGO stage, %** IA–IB IC–II III	26.7% 73.3% 0%	78.3% 13% 8.7%	NA	65.6% 28.1% 6.3%	9.5% 0.5% 0	NA	NA	12 (35.3%) 22 (64.7%) 0 (0%)
**Relapse, *n (%)***	11 (37%)	3 (13%)	12 (12%)	20 (63%)	3 (7%)	2 (4%)	6 (12%)	11 (32%)
**Time to relapse, months (mean/median, IR/ SD)**	8 [4–15] *	29 [8–60] *	25 ± 18.2 **	BC: 16.2 [3–36] * UC + CA: 48 [18–72] *	38.7 [24–60] *	52.5 [31–74] *	29.5 [10–69] *	21.4 [14.3–42.1]
**Type of surgery, %** Cystectomy Adnexectomy	63% 27%	47.8% 52.2%	57.3% 42.7%	100% 0%	28.6% 52.4%	NA	42% 58%	55.9% 44.1%
**Size, cm (mean/median, IR/SD)**	UA: 10 [3–18] * UC, BC: 4.7 [3–8]*	5 [2–12] *	7.5 ± 4.1 **	4.2 [1.2–8] *	6.5 [1.3–11.8] *	NA	UA: 14.9 [10–20] * UC, BC: 8.3 [6.2–12.7]*	8.8 ± 6.8 ** UA: 10.2 ± 9.4 ** UC, BC, UC+CA: 7.7 ± 3.6 **
**Capsular rupture, *n* (%)**	53%	52.2%	33.9%	NA	14%	NA	NA	18 (52.9%)
**Port-site metastases, *n* (%)**	0%	2 (8.7%)	0%	0%	0%	0%	0%	0%
**Endobag use, %**	NA	52.2%	47.%	NA	NA	NA	NA	18 (52.9%)

* Median ± Interquartile Range; ** Mean ± SD. NA: not applicable; BC: bilateral cystectomy; UC: unilateral cystectomy; UA: unilateral adnexectomy; CA: contralateral adnexectomy.

## Data Availability

Original contributions of this study are included in the article/Supplementary Material; further inquiries can be directed to the corresponding author.

## Supplementary Materials

The following supporting information can be downloaded at: https://www.mdpi.com/article/10.3390/jcm13185458/s1, Table S1: Characteristics of oncological outcomes; Table S2: Oncological outcomes of patients with recurrences.
