# Supplementary material for: Laparoscopic Fertility-Sparing Management of Borderline Ovarian Tumors: Surgical and Long-Term Oncological Outcomes
Source: J Clin Med. 2024 Sep 14;13(18):5458. doi: 10.3390/jcm13185458 (PMC11432542; doi:10.3390/jcm13185458)
Supplement: Supplementary file 1 [file jcm-13-05458-s001.zip › Supplemental table S1..pdf]

Supplemental table 1. Characteristics of the oncological outcomes				
	**ALL**	**ADNEXECTOMY**	**CYSTECTOMY**	**P-value**
**Histology, *n (%)***				**1**
Serous	8 (72.7%)	4 (80.0%)	4 (66.6%)	
Mucinous	2 (18.2%)	1 (20.0%)	1 (16.7%)	
Seromucinous	1 (9.1%)	0 (0.0%)	1 (16.7%)	
**FIGO stage, *n (%)***				**0.39**
IA- IB	5 (45.5%)	3 (60.0%)	2 (33.3%)	
IC - II	6 (54.5%)	2 (40.0%)	4 (66.7%)	
**Size, *cm (mean ± SD)***	**5.6 ± 1.4**	**5.6 ± 1.8**	**5.7 ± 1.2**	**0.70**
<10cm	11 (100%)	5 (100%)	6 (100%)	
>10cm	0 (0%)	0 (0%)	0 (0%)	
**Capsular rupture, *n (%)***				**0.04**
Yes	6 (54.5%)	1 (20.0%)	5 (71.4%)	
No	5 (45.5%)	4 (80.0%)	2 (28.6%)	
**Endobag use, *n (%)***				**0.61**
Yes	6 (54.5%)	3 (60.0%)	3 (50.0%)	
No	5 (45.5%)	2 (40.0%)	3 (50.0%)	
**Location of the recidive, *n (%)***				**0.27**
Ovary	11 (100%)	11 (100%)	11 (100%)	
Other	0 (0.0%)	0 (0.0%)	0 (0.0%)	
Staging, n (%)				**0.20**
Yes	4 (36.4%)	3 (60.0%)	1 (20.0%)	
No	7 (63.6%)	2 (40.0%)	4 (80.0%)	
Port-site metastases, n (%)				
Yes	0 (0%)	0 (0%)	0 (0%)	
No	11 (100%)	11 (100%)	11 (100%)	
