# Supplementary material for: Laparoscopic Fertility-Sparing Management of Borderline Ovarian Tumors: Surgical and Long-Term Oncological Outcomes
Source: J Clin Med. 2024 Sep 14;13(18):5458. doi: 10.3390/jcm13185458 (PMC11432542; doi:10.3390/jcm13185458)
Supplement: Supplementary file 1 [file jcm-13-05458-s001.zip › Supplemental table S2..pdf]

Supplemental table 2. Oncological outcomes of patients with recurrences							
	Age	Histology	Surgical approach	FIGO stage	Time to 1st relapse (months)<br><br>Histology<br><br>Treatment	Time to 2nd relapse (months)<br><br>Histology<br><br>Treatment	Total follow-up<br><br>(months)
**1**	35	Mucinous	UC	IC1	23<br><br>Mucinous<br><br>Laparoscopic UA		28
**2**	26	Serous	UA	IIB	16<br><br>Serous<br><br>Laparotomic UA		56
**3**	31	Serous	UA	IA	22<br><br>Serous<br><br>Laparoscopic UC	51<br><br>Serous<br><br>Laparoscopic UA	196
**4**	35	Serous	UA	IA	115<br><br>Serous<br><br>Laparoscopic UC		212
**5**	21	Serous	BC	IC1	28<br><br>Serous<br><br>Laparoscopic UC	77<br><br>Serous<br><br>Laparoscopic UA	287
**6**	36	Mucinous	UA	IA	70<br><br>Mucinous<br><br>Laparoscopic UC		263
**7**	32	Serous	UC	IA	7<br><br>Serous<br><br>Laparoscopic UC		72
**8**	27	Serous	UC	IC1	16<br><br>Serous<br><br>Laparoscopic BC		168
**9**	40	Serous	UA	IC3	45<br><br>Serous<br><br>Laparoscopic UC		114
**10**	38	Serous	UC	IA	37	12	115

					Serous<br><br>Laparoscopic UA	Serous<br><br>Laparoscopic histerectomy + UA	
**11**	38	Serous-mucinous	BC	IC1	18<br><br>Mucinous<br><br>Laparoscopic BA		132
**BC: Bilateral cystectomy; UC: Unilateral cystectomy; UA: Unilateral adnexectomy**							
